# Syndrome coronaire aigu: un mode de révélation peu fréquent du phéochromocytome

**DOI:** 10.11604/pamj.2015.22.151.7505

**Published:** 2015-10-16

**Authors:** Abderrahim El Bouazzaoui, Nawal Hammas, Nawfal Houari, Brahim Boukatta, Abdelmalek Oussaden, Hicham Sbai, Nabil Kanjaa

**Affiliations:** 1 Service d'Anesthésie Réanimation A4, CHU Hassan II, Faculté de Médecine et de Pharmacie, Université Sidi Mohammed Benabdellah (USMBA), Fès, Maroc; 2 Service d'Anatomie Pathologique, CHU Hassan II, Faculté de Médecine et de Pharmacie, Université Sidi Mohammed Benabdellah (USMBA), Fès, Maroc; 3 Service de Chirurgie Viscérale C3, CHU Hassan II, Faculté de Médecine et de Pharmacie, Université Sidi Mohammed Benabdellah (USMBA), Fès, Maroc

**Keywords:** Phéochromocytome, syndrome coronaire, coronarographie, Pheochromocytoma, coronary syndrome, coronary angiography

## Abstract

Le phéochromocytome est une tumeur rare. Il se manifeste habituellement par la triade de Ménard ou par une hypertension artérielle. Des cas rares de phéochromocytome ont été révélés par un syndrome coronaire aigu. Patiente âgée de 57 ans, ayant l'antécédent d'hypertension artérielle sous amlodipine, est admise pour syndrome coronaire aigue sans sus décalage du segment ST à troponine positive. La coronarographie est revenue en faveur d'un réseau coronaire grêle sans sténose significative. Une masse tissulaire surrénalienne droite a été retrouvée sur une échographie rénale de routine et confirmée par un scanner scanner abdominal. Le dosage des cathecholamines urinaires trouve des taux très élevés d'adrénaline et de noradrenaline. Apres une préparation par un β bloquants associé à un inhibiteur calcique, l'exérèse chirurgicale de la tumeur a été réalisée par voie cœlioscopique sous monitorage hémodynamique invasif. L'examen anatomopathologique de la pièce opératoire confirme le diagnostic de phéochromocytome. L’évolution à court et moyen terme est favorable, notamment disparition de la triade de Menard, des douleurs thoraciques. La révélation d'un phéochromocytome par la survenue d'un syndrome coronaire aigu est rare, souvent ignorées, mais dont les conséquences peuvent être dramatiques. La tumorectomie peut conduire à la guérison complète et définitive des manifestations cardiaques.

## Introduction

Le phéochromocytome est une tumeur rare composée de cellules chromaffines sécrétant des catécholamines et localisée au niveau de la médullosurrénale dans 85% des cas. Le diagnostic est évoqué le plus souvent dans le cadre du bilan d'une hypertension artérielle ou devant la triade symptomatique de Ménard [1]: céphalées, sueurs et palpitations chez un adulte de 15 à 40 ans. Plus rarement, les manifestations cardiovasculaires du phéochromocytome aboutissent à son diagnostic (trouble du rythme, douleur thoracique, insuffisance cardiaque, choc cardiogénique) [2]. Nous rapportons l'observation d'une patiente âgée de 57 ans dont le phéochromocytome a été révélé par un syndrome coronaire aigu.

## Patient et observation

**Figure 1:**
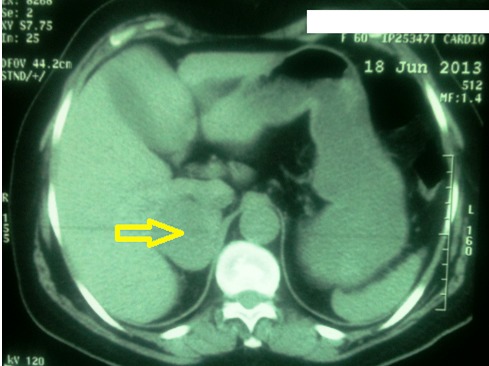
Masse surrénalienne droite de 50 mm de diamètre (flèche jaune) dont le volume, la densité et l'hétérogénéité peuvent être évocateurs de phéochromocytome

**Figure 2:**
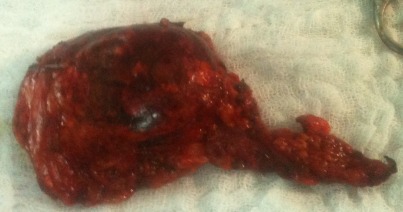
Photo de pièce opératoire de surrenalectomie

**Figure 3:**
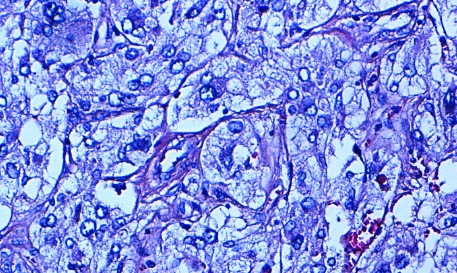
HESx200: phéochromocytome: prolifération tumorale faite de nids de cellules arrondies avec une vascularisation de type neuro-endocrine

Patiente âgée de 57 ans ayant comme facteurs de risque cardiovasculaire l’âge, la ménopause, et l'hypertension artérielle sous amlodipine (10 mg/j). Elle a été admise à l'hôpital dans un tableau de syndrome coronaire aigu. Le début de sa symptomatologie remontait à 3 jours par la survenue de douleurs thoraciques constrictives au repos, sans irradiation, brèves et intermittentes sans relation avec l'effort. A l'examen cardiovasculaire on note une tension artérielle à 170/90 mmHg, avec une fréquence cardiaque à 80 battements par minute, sans signes d'insuffisance cardiaque. Les pouls périphériques sont présents et symétriques. L'auscultation des artères carotidiennes ne trouve pas de souffle. L'examen peluropulmonaire est sans anomalie. L’électrocardiogramme s'inscrit en rythme sinusal avec une FC à 73 cycles par minute, un PR à 0.20, un axe du cœur à gauche. On note la présence d'onde T négatives avec un sous décalage du segment ST en antérieur étendu, en inferieur et en basal avec des extrasystoles supra ventriculaires. Le dosage de la troponine est revenu positive à 2 reprises à 12 heures d'intervalle. La radiographie thoracique objective une cardiomégalie avec un index cardiothoracique à 0,63. L'analyse du parenchyme pulmonaire trouve une surcharge hilaire bilatérale avec une redistribution vasculaire vers les sommets. L’échocardiographie transthoracique objective un ventricule gauche hypertrophié à fonction systolique normale, la contractilité segmentaire est hétérogènes avec une hypokinesie latérale. Les pressions de remplissage sont élevées, les cavités droites ne sont pas dilatées sans hypertension artérielle pulmonaire. La coronarographie objective un réseau coronaire grêle avec une plaque non significative sur l'artère coronaire inter ventriculaire antérieure. Devant ce tableau de syndrome coronaire aigue sans sus décalage du segment ST, la patiente a été mise sous traitement médical comportant une anti coagulation curative, une double anti agrégation plaquettaire associant aspirine avec clopidogrel, bétabloquant, inhibiteur de l'enzyme de conversion, et une statine. Au bilan biologique on trouve une fonction rénale altéré (urée 0,72 g/l, créatinémie à 20 mg/l avec une clairance à 40 ml/min). La natrémie et la kaliémie sont normales. Le bilan lipidique est normal. La numération formule sanguine est normale avec un taux de plaquettes à 236000/mm^3^, et une hémoglobine à 14 g/l. Devant cette fonction rénale altérée, une échographie rénale a été réalisée montrant des reins de taille normale sans dilatation des voies excrétrices, et objectivant une masse tissulaire surrénalienne droite. Le retour à l'interrogatoire retrouve la notion d'accès paroxystique de palpitation, sueurs et céphalées. Un scanner abdominal a été réalisé objectivant une masse surrénalienne droite de 50 mm de diamètre faisant évoquer un phéochromocytome (Figure 1). Le dosage des cathecholamines urinaires trouve des taux très élevés d'adrénaline et de noradrenaline: 8,68 micromol/ 24h et 3,51 µmol/ 24h respectivement. L’échographie cervicale ne révèle pas de lésions thyroïdiennes. Apres une préparation par un α bloquants associé à un inhibiteur calcique, l'exérèse chirurgicale de la tumeur (Figure 2) a été réalisée par voie c'lioscopique sous monitorage hémodynamique invasif. Des pics hypertensif arrivant jusqu’à 220 mmHg de systolique et 130 mmHg de diastolique ont notés au moment de la manipulation chirurgicale de la tumeur. Ces pics ont été jugulés par l'approfondissement de l'anesthésie associé à des bouli itératifs de nicardipine. Les suites postopératoires ont été marquées par la stabilité des chiffres tensionnels sous inhibiteur calcique en monothérapie. L'examen anatomopathologique de la pièce opératoire confirme le diagnostic de phéochromocytome (Figure 3). L’évolution à court et moyen terme est favorable chez notre patiente avec disparition de la triade de Menard, des douleurs thoraciques sous traitement médical associant statine, aspirine et l'amlidipine à 5 mg/j.

## Discussion

Le phéochromocytome est une tumeur développée à partir des cellules chromaffines de la médullosurrénale ou d'autres ganglions sympathiques et sécrétant en quantité variable des cathécholamines. Le diagnostic est évoqué le plus souvent dans le cadre du bilan d'une hypertension artérielle ou devant la triade symptomatique de Ménard. Au pronostic souvent favorable après son exérèse, cette tumeur peut aussi être à l'origine de complications cardiovasculaires parfois dramatiques. La complication la plus classique est l'hypertrophie ventriculaire gauche secondaire à l'HTA, pouvant donner une véritable cardiomyopathie hypertrophique obstructive. La cardiomyopathie adrénergique, quant à elle, est moins connue. Elle a été initialement suspectée par Pearce en 1906 [3] qui a mis en évidence des lésions de myocardite sur des animaux morts après injection d'adrénaline, ces mêmes lésions ont été retrouvées par la suite chez des malades traités par adrénaline pour des états de choc ou atteints de phéochromocytome. Le syndrome coronaire aigu reste un mode de révélation rare de phéochromocytome [4–6]. Le diagnostic de phéochromocytome méconnu peut être difficile dans ce contexte. En effet, Delby et al ont rapporté une découverte tardive de phéochromocytome chez deux patients ayant bénéficié de transplantation cardiaque dont l'indication initiale était une cardiomyopathie dilatée d'origine ischémique [7]. Dans ces conditions atypique on considère que 50% des phéochromocytomes sont diagnostiqués en période post mortem [8]. Les mécanismes physiopathologiques de la cardiomyopathie adrénergique associée au phéochromocytome restent flous, faisant intriquer plusieurs hypothèses [4, 9–11]: l'hypothèse d'une insuffisance coronaire de type fonctionnelle par épuisement des réserves énergétiques sur un c'ur imprégné de façon chronique par les catécholamines est avancée; lors des paroxysmes hypertensifs, il se produit un largage massif de catécholamines, responsable d'une tachycardie et donc d'un accroissement brutal des besoins du myocarde en oxygène; d'autres hypothèses telles qu'une insuffisance coronaire de type organique liée à la déstabilisation de lésion athéromateuse par la stimulation adrénergique ou encore un éventuel spasme des gros troncs coronaires ont été avancées pour expliquer ces tableaux mimant parfois un véritable infarctus.

Électriquement, les aspects sont variables: lésion sous-épi ou sous-endocardique, ischémie sous épicardique, onde Q de nécrose,…. Les signes prédominent souvent dans le territoire inférolatéral. Les enzymes myocardiques peuvent s’élever. Les coronaires sont angiographiquement saines. Les formes graves de phéochromocytome sont souvent associées à des nécroses hémorragiques de la tumeur responsable d'orage catécholaminérgique [6]. Cependant, d'autres facteurs déclenchant ont été rapportés [12]: stress non spécifique, ingestion d'alcool, grossesse, palpation ou traumatisme abdominal. Certains de ces facteurs sont iatrogènes comme la prise de métoclopramide, dopaminergiques, propanolol, sultopride, glucagon, phénothiazines, dexaméthasone. Ces orages hémodynamiques peuvent être responsables d'autres modes de révélations parfois graves en dehors des complications cardiaques. Nous en citons: accident vasculaire, infarctus mésentérique, ischémie de membre, ou encore rhabdomyolyse aigue [13]. Le traitement curatif repose sur l'exérèse chirurgicale de la tumeur [14]. Cette exérèse peut se concevoir en urgence dans les cas ou’ le phéochromocytome est associé à un infarctus de myocarde compliqué de choc cardiogénique [15]. Notre patiente était stable sur le plan hémodynamique. L'exérèse chirurgicale de la tumeur a été réalisée en dehors du contexte d'urgence.

## Conclusion

Le phéochromocytome peut avoir des présentations cliniques très variables. La révélation d'un phéochromocytome par la survenue d'un syndrome coronaire aigu est rare, souvent ignorées, mais dont les conséquences sont parfois dramatiques. Cela est d'autant plus important que la tumorectomie seule peut conduire à la guérison complète et définitive des manifestations cardiaques.
